# Uniportal video-assisted thoracoscopic surgery lobectomy in Bahrain: a case report

**DOI:** 10.1093/jscr/rjac251

**Published:** 2022-07-08

**Authors:** Sundus AlMukhodher, Mariam Asheer, Ghassan Alfaqaawi, Osama Bader

**Affiliations:** Department of Surgery, Salmaniya Medical Complex, Bahrain; Department of Surgery, Salmaniya Medical Complex, Bahrain; Department of Surgery, Salmaniya Medical Complex, Bahrain; Department of Surgery, Salmaniya Medical Complex, Bahrain

## Abstract

Uniportal video-assisted thoracoscopic surgery (VATS) is minimally invasive thoracic surgery that does not use a formal thoracotomy incision. It was first introduced by Dr Diego Gonzalez-Rivas in 2011. We report here our first case performed in Salmaniya Medical Complex in Bahrain using uniportal VATS lobectomy for a patient with colorectal cancer who had a lung metastasis. Uniportal VATS lobectomy is a safe and feasible procedure when performed by an experienced surgeon. It has remarkably reduced postoperative complications as well as the length of stay. However, survival percentage VATS lobectomy appears to be equivalent to survival percentage obtained with open lobectomy.

## INTRODUCTION

After more than two decades of advancements, video-assisted thoracoscopic surgery (VATS) has become the gold standard for treating lung cancer around the world. The change from conventional multi-port VATS to the use of just a single port seems like such a radical step that many have viewed it as perhaps the single greatest leap forward in minimally invasive thoracic surgery since the birth of VATS itself [1]. Indeed, many have described it as ‘revolutionary’, and its benefits are under investigation. Several recent trials of uniportal VATS pulmonary resection have revealed diminished postoperative discomfort and paresthesias, and some surgeons have even noted benefits including improved equipment design and exposure during operation [2]. The first uniportal VATS lobectomy with mediastinal lymph nodes dissection was done in 2011 by the expert Dr Gonzalez-Rivas. Since then, this new technique has become a trend all over the world [3]. In May 2019, VATS lobectomy was successfully done for the very first time in our hospital.

## CASE PRESENTATION

We report a 62-year-old Bahraini male ex-smoker, with a known history of hypertension and colon cancer stage IV with liver and lung metastases. In 2012, he received 30 cycles of neoadjuvant chemotherapy. He was then operated and 10 cm of the colon was resected followed by 9 cycles of chemotherapy. During the computed tomography (CT) scan follow-up, liver metastases were found and operation was done for segmental liver resection in 2017 and received 12 cycles of chemotherapy. During follow up, CT scan was performed and an Incidental finding was found in the lung in 2019. He was admitted for further investigations and treatment.

**Figure 1:**
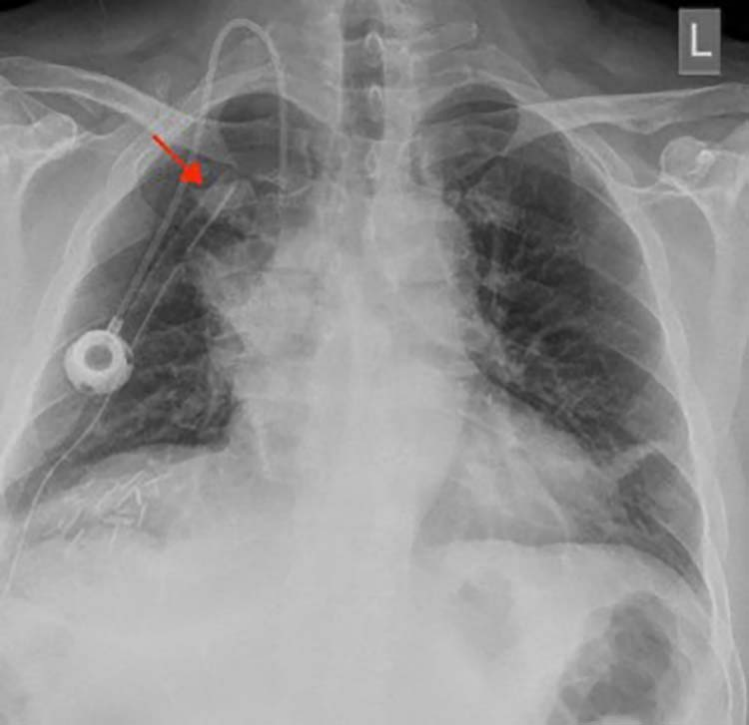
Chest X-ray (PA view) showing the right upper lobe mass. PA: posterior anterior.

**Figure 2:**
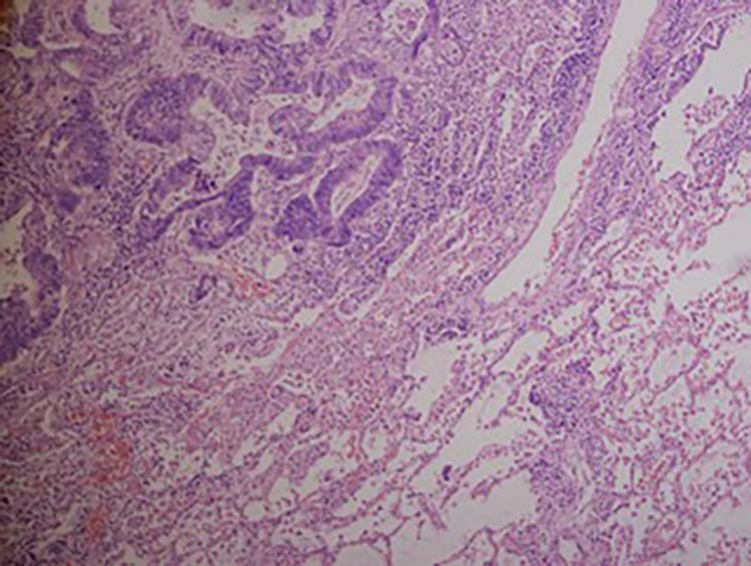
Hematoxylin and eosin stained histopathologic images illustrating lung tissue infiltrated by metastatic adenocarcinoma from colon (hematoxylin–eosin, original magnification ×200).

**Figure 3:**
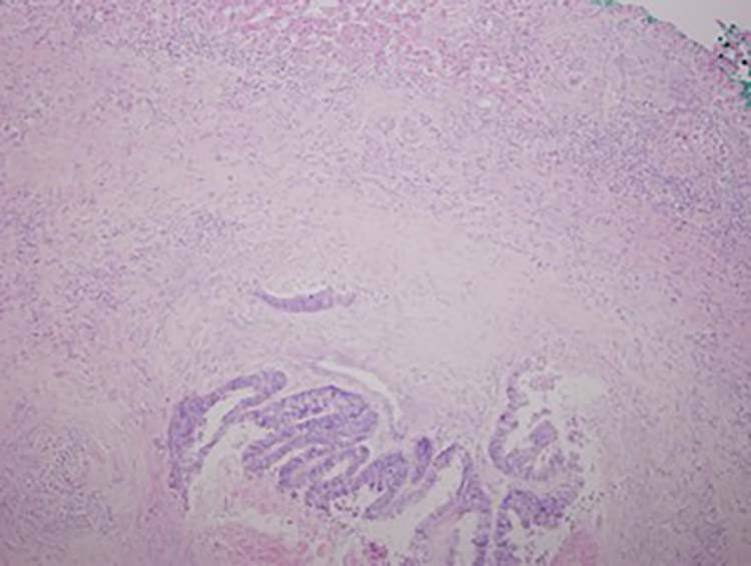
Low magnification power view of liver tissue with metastatic deposit from colon adenocarcinoma. (hematoxylin–eosin original magnification ×100).

A chest X-ray at the time of admission showed right upper lobe mass (Fig. 1). Tru-Cut biopsy was performed and confirmed the diagnosis of non-small cell carcinoma (Figs 2 and 3). Multiple sections showed two nodules composed of well differentiated adenocarcinoma. The largest one measuring 4 mm from the resection margin, 2.5-cm away from hilar region and 5 mm from the outer surface. Smallest nodule was also composed of well-differentiated adenocarcinoma present 3 mm from the outer surface.

A 5-cm single incision was made between anterior and middle axillary line at the level of fourth intercostal space. Initially, the superior pulmonary vein identified at the right upper lobe and sacrificed using ‘TAN GIA’ stapler and the posterior port of oblique fissure noted to be completed. Then the truncus anterior and posterior ascending artery were identified and sacrificed using ’TAN GIA’ stapler. The right upper lobe bronchus identified and stapled using ‘endo GIA’ tristapler and the right paratracheal lymph node was dissected and removed. Finally, a 28 Fr chest tube was inserted through the same incision, and the chest wall closed in layers with 3/o vicryl interrupted for the muscles and clips for the skin.

## DISCUSSION

The long-term goals of lung cancer surgery include improved length and quality of life. As a result of this increased experience and comfort with VATS lobectomy, thoracic surgeons have implemented its principles in several subsets of lung cancer patients who would not have got a curative resection or would have required a thoracotomy [4]. VATS wedge resection can be an appropriate choice for patients with poor pulmonary function, advanced age and tiny peripheral tumors who cannot endure lobectomy or thoracotomy [5, 6].

VATS lobectomy patients are observed to have fewer serious sequelae than open lobectomy patients, including atrial fibrillation, atelectasis, persistent air leak, pneumonia and renal failure. The VATS lobectomy group presents with a shorter chest tube stay and a shorter hospital stay [2, 7].

The 30-degree telescope is preferred in traditional VATS surgery for two reasons: first, the visual port limits the telescope lens’s moving range; second, the telescope’s direction differs significantly from that of the instruments, so turning the 30-degree lens will align the visual plane with the operating plane. These two issues are not present in uniportal VATS techniques. Because the telescope enters a larger incision, it has greater mobility; and because the telescope’s direction is nearly identical to that of the instruments, the visible plane and operating plane do not have to coincide, the lens does not need to be moved. Furthermore, a 0-degree telescope must be hoisted around 30 degrees higher than a 30-degree telescope to achieve the same vision angle, which leaves more space for the instrumentation to enter below the telescope lens. Another benefit of 0-degree telescopes is that they are considerably easier to control with one hand, allowing the helper to assist in exploration with the other. All uniportal VATS lung resections, including wedge resection, lobectomy and mediastinal lymph node dissection, should, in our opinion, be performed using a 0-degree telescope [8]. Because the incisions are much smaller and the telescopes will intrude much deeper into the thoracic cavity for procedures like thymectomy, bullectomy and sympathectomy, the telescope’s mobility will be limited, as it is with traditional (2–3 port) VATS procedures, for which a 30-degree telescope will be preferable [9].

In uniportal VATS lobectomy, the staplers used are crucial. The ones with articulation would be chosen because they allow more flexibility while being positioned via particular angles of anatomic structures. The new staplers with the curved tip at the anvil’s distal end can enable better visibility and mobility around the target vessels. Endoscopic staplers without articulation were employed in this case. The surgery can still be done with staplers without articulation with the surgeon’s sensitive and thorough separation of the hilar anatomical structure and cautious manoeuvring with the staplers.

## CONCLUSIONS

Uniportal VATS lobectomy is a safe and feasible procedure when performed by an experienced surgeon. It has remarkably reduced postoperative complications as well as the length of stay. However, survival percentage VATS lobectomy appears to be equivalent to survival percentage obtained with open lobectomy [10].
